# Fatty liver is a risk factor for liver metastasis in Chinese patients with non-small cell lung cancer

**DOI:** 10.7717/peerj.6612

**Published:** 2019-03-14

**Authors:** Wenyu Wu, Haiyan Liao, Weilin Ye, Xi Li, Jian Zhang, Junguo Bu

**Affiliations:** 1 Department of Oncology, Zhujiang Hospital, Southern Medical University, Guangzhou, China; 2 Target and Interventional Therapy Department of Oncology, First People’s Hospital of Foshan, Foshan, China; 3 Department of Radiation Oncology, Zhujiang Hospital, Southern Medical University, Guangzhou, China

**Keywords:** Non-small cell lung cancer, Hepatic steatosis, Liver metastasis

## Abstract

**Background:**

The hepatic microenvironment, which may include chronic inflammation and fibrosis, is considered to contribute to the development of liver metastases. Hepatic steatosis (HS) might cause liver inflammation and fibrosis. However, to date, no studies have investigated the impact of HS on liver metastasis in patients with non-small cell lung cancer (NSCLC).

**Methods:**

A retrospective cohort study was performed on patients who received NSCLC treatment at two hospitals affiliated with the Southern Medical University from January 2005 to December 2015. The patients were grouped according to the presence of HS. The clinicopathological features of patients between the two groups were compared. The effect of HS on liver metastasis and overall metastasis was evaluated, adjusting for other confounders using Cox regression analyses.

**Results:**

In total, 1,873 patients with NSCLC with no distant metastases were included in this study, and 408 (21.8%) patients were diagnosed with HS (at the time of diagnosis or before diagnosis). Liver metastases occurred in 166 (8.9%) patients. Liver metastasis-free survival was significantly worse in the study (HS) group (hazard ratio (HR) 1.42; (95% CI [1.03–1.96]); *P* = 0.031). Multivariate regression analysis demonstrated that HS was an independent risk factor for liver metastasis (HR 1.43; 95% CI [1.02–2.01]; *P* = 0.039). However, HS was not associated with overall metastasis of NSCLC (HR 0.99; 95% CI [0.84–1.17]; *P* = 0.895).

**Conclusion:**

Hepatic steatosis was an independent predictor of liver metastasis from in patients with NSCLC.

## Introduction

Non-small cell lung cancer (NSCLC) accounts for approximately 85% of lung cancer, and distant organ metastasis is the leading cause of death in these patients, with a 5-year survival rate of less than 15% (Siegel, Miller & Jemal, 2016; Wang et al., 2010). In advanced-stage lung cancer, metastasis sites of distant organs, including brain, bone and liver, are common (Riihimaki et al., 2014). Among the distant metastases, liver metastasis in NSCLC patients confers the worst prognosis, even worse than brain metastases, with a median overall survival of 3 months (Riihimaki et al., 2014; Tang et al., 2016). Despite recent advances in various treatment strategies, no local consolidation therapies of liver metastases have been established in clinical practices (Jiang et al., 2017; Langley & Fidler, 2011). Therefore, increased knowledge of the risk factors for liver metastasis may be an effective strategy for lowering the incidence of this lethal disease.

Non-alcoholic fatty liver disease (NAFLD) is one of the most common hepatic disorders worldwide, and is significantly associated with obesity and age (Bellentani, 2017). Currently, the population prevalence of NAFLD in Asia is approximately 25%, comparable to Western countries (Fan, Kim & Wong, 2017). Therefore, NAFLD is commonly encountered in many cancer patients, such as NSCLC patients, in clinical oncology practices. The histological hallmark of NAFLD is hepatic steatosis (HS), which can lead to a liver lesion that is characterized by hepatocyte injury and inflammation changes with or without fibrosis (Hardy et al., 2016). Given that the poor prognosis of NSCLC patients with liver metastasis and NAFLD are endemic worldwide, it is important to clarify the association between HS and liver metastasis in NSCLC patients.

Recently, opinions abound on whether liver metastasis could be affected by HS have no consistent conclusion. In patients with colorectal cancer, two observational studies just reported conflicting results. Hamady et al. (2013) suggested that HS was an independent risk factor for liver metastasis in patients with colorectal cancer. On other hand, Murono et al. (2013) reported that liver metastases derived from colorectal cancer occur less frequently in patients with HS. In breast cancer, it has been reported that liver metastases rarely occur in patients with HS (Wu et al., 2017).

However, to the best of our knowledge, no study to date has investigated the relationship between HS and liver metastasis in patients with NSCLC. Therefore, the objective of the current two-center cohort study was to determine whether liver metastasis of NSCLC patients with no distant metastases could be influenced by HS.

## Materials and Methods

### Study population

From January 1, 2005 to December 31, 2015, we conducted this two-center cohort study of patients with NSCLC, at two hospitals affiliated with the Southern Medical University (Guangzhou, China), Nanfang Hospital and Zhujiang Hospital. In total, 1,873 patients with new pathologically diagnosed NSCLC were enrolled in this two-center retrospective study. The clinicopathological information, including sex, age, date of NSCLC diagnosis, date of liver metastasis, tumor histology, treatment and laboratory data, was reviewed from hospital medical records. The clinical staging was determined based on the tumor, node, metastasis classification of the AJCC system (7th edition, 2010).

The inclusion criteria were as follows: (1) patients with histopathologically confirmed NSCLC, (2) patients who had undergone an abdominal ultrasound (US) within a month of the NSCLC diagnosis, and (3) patients who did not consume excessive amounts of alcohol.

The exclusion criteria were as follows: (1) patients with distant metastases at that time of the NSCLC diagnosis, (2) patients with other malignant tumors, and (3) patients with follow-up times of fewer than 30 days.

The study protocol protected the patients’ private information according to the precepts of the Helsinki Declaration. Additionally, approvals were acquired from the Institutional Review Boards of the Nanfang Hospital and Zhujiang Hospital of Southern Medical University (2017-zlk-009).

### Laboratory tests

As hepatitis B virus is prevalent in China, the sera of all patients were screened for hepatitis B surface antibody and hepatitis C virus antibody using ELISA (enzyme-linked immunosorbent assay). In addition, patients who underwent radiotherapy or chemotherapy routinely received liver function tests, including aspartate aminotransferase (AST), alanine aminotransferase (ALT), and total bilirubin levels every 2 weeks during treatment or at each follow-up visit.

### Imaging evaluation of the liver, metastasis assessment, follow-up and treatment

Compared with pathological biopsy as a standard method, US was found to have high specificity and sensitivity for the detection of fatty liver (Dasarathy et al., 2009; Hamaguchi et al., 2007); all patients treated in the two study hospitals routinely had an US to check for liver diseases at that time of diagnosis or during follow-ups.

In accordance with the guidelines for the assessment and management of NAFLD in the Asia-Pacific region (Review Team et al., 2014), the diagnosis of NAFLD can be based on the following factors: (1) the imaging results are consistent with the diagnostic criteria of fatty liver disease, in which is a liver fat content of >5% is considered diagnostic of NAFLD, (2) no history of alcohol intake more than 30 g for males or 20 g for females per day, and (3) exclusion of other diseases that can lead to steatosis.

Based on the abdominal US results, patients were divided into the study group and control group. Therefore, the patients with HS were included in the study (HS) group, and others without HS were regarded as in the control (non-HS) group.

All patients underwent routine imaging assessments, including abdominal US or computed tomography (CT), thoracic radiography, CT, or magnetic resonance imaging (MRI), brain CT and whole-body bone scans at the time of diagnosis or during follow-ups, and their metastatic status was evaluated by imaging specialists. Routine abdominal US were performed during follow-up, and if the results showed a potential liver metastasis, further validation was performed via CT or MRI. Alternatively, the liver metastasis was diagnosed directly by CT or MRI in the absence of US. All imaging records were evaluated separately by two specialized radiologists to increase the accuracy of metastatic status diagnosis; disagreements were resolved by consensus.

The duration of follow-ups was calculated from the day of the histopathological biopsy to the date of diagnosis of liver metastasis, or to the last imaging examination. The formulation of the follow-up protocols and treatments were implied referring to the NCCN Clinical Practice Guidelines of NSCLC. The last follow-up date was December 31, 2017.

### Statistical analysis

We compared the clinicopathological factors in two groups using χ^2^ tests or Fisher’s exact tests, as indicated. The Kaplan–Meier method was used to calculate liver and overall metastasis-free survival (MFS) rates, and the differences in survival for univariate comparisons were calculated by using the log-rank test. To determine the potential effect modification by age, gender, ALT or AST level, hepatitis virus, diabetes mellitus, and BMI, an interaction analysis was calculated by adding the respective categorical variable product terms individually to the model. A Cox regression analysis was used to analyze univariate and multivariate analyses for liver and overall metastasis. Hazard ratios (HRs) with 95% confidence intervals (CIs) were used to show the results.

SPSS statistical software (version 20; IBM Corporation, Chicago, IL, USA) was used for all analyses. The criterion for statistical significance was determined by two-tailed *P*-values less than 0.05.

## Results

### Baseline parameters of NSCLC patients

A total of 1,873 patients (878 from Nanfang Hospital and 995 from Zhujiang Hospital) were conformed for the analysis during the study period. A total of 408 (21.8%) patients were diagnosed with HS. Thus, 408 (21.8%) patients with HS were included in the study group, and 1,465 (78.2%) patients were regarded as the control group.

The median follow-up time was 14.5 ± 16.2 months (range, 1–131 months) in the study population. The mean age of all patients was 58.9 ± 11.1 years. The monitored endpoints were the liver and overall MFS rates. There were 762 patients with distant organ metastases. Liver metastases were observed in 58 (14.2%) of 408 patients in the study group and 108 (7.4%) of 1,465 patients in the control group, suggesting that the liver metastasis rate in the study group was significantly higher compared with the control group (*P* < 0.001; χ^2^ test).

Baseline parameters of the study and control groups and differences in the distribution of covariables are listed in Table 1. There were no significant differences in the T classification, N classification, histology, surgery, chemotherapy or radiotherapy between the two groups. However, higher proportions of HS were observed in younger patients (aged <60 years; *P* < 0.001), in male patients (*P* = 0.002), and in patients without the hepatitis virus infection (9.1% vs. 12.8%; *P* = 0.039). Furthermore, HS was found at a higher incidence in obese (BMI ≥ 25 kg/m^2^) patients and patients with diabetes mellitus (*P* < 0.001 and *P* = 0.015, respectively). Moreover, a high level of the ALT or AST was significantly more common in the study group compared with the control group (14.0 vs. 7.8%, *P* < 0.001).

**Table 1:** Baseline characteristics of the NSCLC patients.

Characteristic	Study group (408)	Control group (1,465)	*P*-value
Age			<0.001
<60 years	275 (67.4)	724 (49.4)	
≥60 years	133 (32.6)	741 (50.6)	
Gender			0.002
Male	315 (77.2)	1,015 (69.3)	
Female	93 (22.8)	450 (30.7)	
T category			0.705
T1	52 (12.7)	173 (11.8)	
T2	155 (38.0)	533 (36.4)	
T3	76 (18.6)	309 (21.1)	
T4	125 (30.6)	450 (30.7)	
N category			0.370
N0	105 (25.7)	377 (25.7)	
N1	45 (11.0)	145 (9.9)	
N2	150 (36.8)	601 (41.0)	
N3	108 (26.5)	342 (23.3)	
Pathological type			0.298
Squamous	133 (32.6)	519 (35.4)	
Non-squamous	275 (67.4)	946 (64.6)	
ALT or AST level			<0.001
<40 U/L	351 (86.0)	1,350 (92.2)	
≥40 U/L	57 (14.0)	115 (7.8)	
Hepatitis virus			0.039
Present	37 (9.1)	188 (12.8)	
Absent	371 (90.9)	1,277 (87.2)	
Diabetes mellitus			0.015
Yes	45 (11.0)	107 (7.3)	
No	363 (89.0)	1,358 (92.7)	
BMI			<0.001
<25 kg/m^2^	240 (58.8)	1,183 (80.8)	
≥25 kg/m^2^	168 (41.2)	282 (19.2)	
Surgery			0.235
Yes	157 (38.5)	517 (35.3)	
No	251 (61.5)	948 (64.7)	
Chemotherapy			0.654
Yes	372 (91.2)	1,325 (90.4)	
No	36 (8.8)	140 (9.6)	
Radiotherapy			0.360
Yes	96 (23.5)	328 (22.4)	
No	312 (76.5)	1,137 (77.6)	

**Note:**

ALT, alanine aminotransferase; AST, aspartate aminotransferase; BMI, body mass index.

### Prognostic factors for liver metastasis-free survival

**Figure 1:**
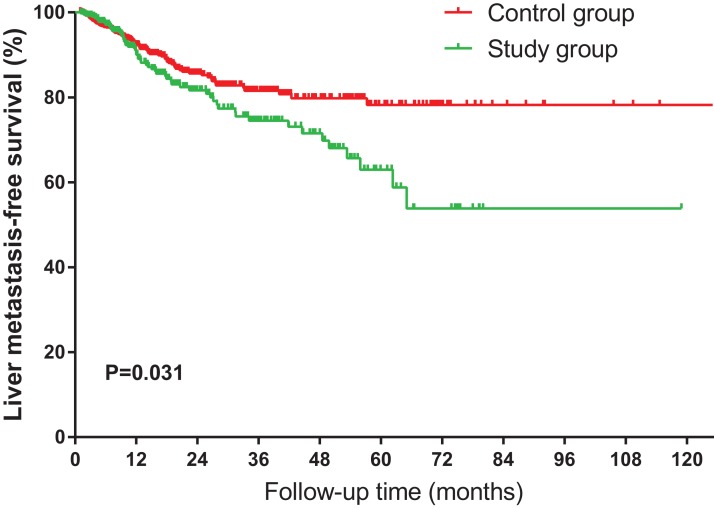
Kaplan–Meier curves of liver metastasis-free survival for NSCLC patients, stratified by HS.

The liver MFS rates are shown in Fig. 1. The 5-year liver MFS rates were 54.1% and 78.7% in the study group and control group, respectively, indicating that patients in the study group had a significantly worse liver MFS (*P* = 0.031). Furthermore, in the univariate Cox regression analysis, HS was associated with poorer liver MFS.

**Figure 2:**
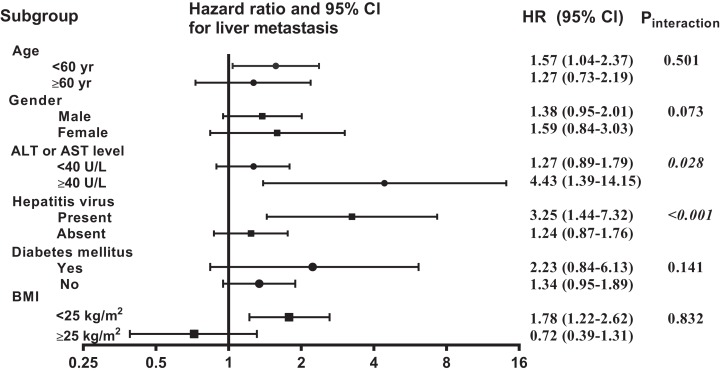
Subgroup analyses of liver metastasis-free survival comparing the study and control populations.

In the multivariate Cox regression analysis adjusted for all confounders, HS was considered an independent adverse factor for liver metastasis (HR 1.43; 95% CI [1.02–2.01]; *P* = 0.039) (Table 2). Moreover, advanced N classification (N1, N2, and N3) was associated with an increased risk of liver metastasis in the NSCLC patients. In contrast, surgery (HR 0.57; 95% CI [0.38–0.84]; *P* = 0.005) and radiotherapy (HR 0.57; 95% CI [0.39–0.83]; *P* = 0.003) were found to be independent protective factors for liver metastasis (Table 2), which is consistent with previous studies. In the subgroup analysis (Fig. 2), there were no significant interactions in age, gender, diabetes mellitus, or BMI, with overall *P*-values of 0.501, 0.073, 0.141, and 0.832, respectively; except for AST or ALT level and hepatitis virus (*P*_interaction_ = 0.028 and *P*_interaction_ < 0.001, respectively).

**Table 2:** Univariate and multivariate analyses for liver metastasis.

Parameter	Univariate analysis		Multivariate analysis	
	HR (95% CI)	*P*-value	HR (95% CI)	*P*-value
Age ≥60 years	1.03 (0.76–1.40)	0.839	0.96 (0.70–1.31)	0.788
Gender: Male	1.18 (0.84–1.66)	0.335	1.22 (0.86–1.74)	0.268
T category				
T1	1		1	
T2	2.23 (1.20–4.15)	0.011	1.93 (1.03–3.61)	0.042
T3	2.20 (1.12–4.33)	0.022	1.41 (0.70–2.82)	0.335
T4	3.02 (1.62–5.63)	<0.001	1.77 (0.93–3.39)	0.082
N category				
N0	1		1	
N1	1.10 (0.51–2.39)	0.817	1.01 (0.46–2.21)	0.990
N2	3.10 (1.92–4.99)	<0.001	2.71 (1.65–4.47)	<0.001
N3	4.52 (2.75–7.42)	<0.001	3.59 (2.10–6.13)	<0.001
Histology squamous	1.27 (0.93–1.74)	0.140	1.28 (0.93–1.77)	0.140
ALT or AST ≥40 U/L	0.97 (0.57–1.66)	0.923	0.81 (0.47–1.42)	0.467
Hepatitis virus	1.25 (0.81–1.93)	0.306	1.30 (0.83–2.04)	0.246
Diabetes mellitus	1.10 (0.66–1.85)	0.707	1.12 (0.67–1.90)	0.662
BMI ≥25 kg/m^2^	1.27 (0.90–1.78)	0.177	1.07 (0.75–1.53)	0.709
Hepatic Steatosis	1.42 (1.03–1.96)	0.032	1.43 (1.02–2.01)	0.039
Surgery	0.45 (0.31–0.63)	<0.001	0.57 (0.38–0.84)	0.005
Chemotherapy	1.21 (0.20–2.09)	0.504	0.76 (0.43–1.34)	0.346
Radiotherapy	0.72 (0.50–1.05)	0.087	0.57 (0.39–0.83)	0.003

**Note:**

ALT, alanine aminotransferase; AST, aspartate aminotransferase; BMI, body mass index; HR, hazard ratio; CI, confidence interval.

**Figure 3:**
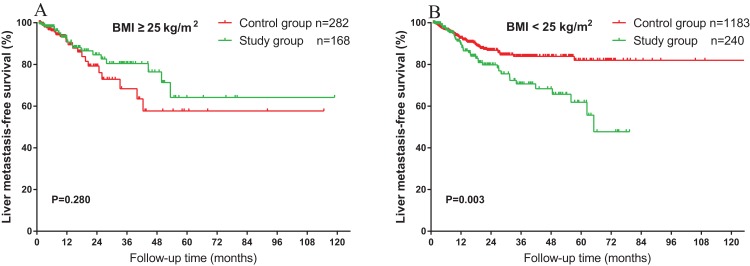
Impact of BMI on the association between HS and liver metastasis. (A) No significant differences of liver MFS were observed between the patients with and without HS among the overweight patients. (B) Non-overweight (BMI <25 kg/m^2^) patients with HS had significantly worse liver MFS (*P* = 0.003) compared to those without HS.

**Figure 4:**
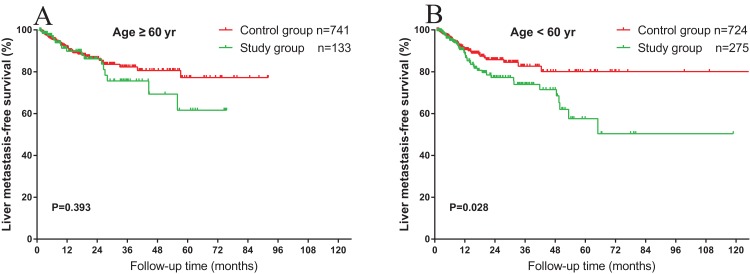
Impact of age on the association between HS and liver metastasis. (A) No significant differences of liver MFS were observed between patients with and without HS among the older patients. (B) Younger (age <60 years) patients with HS had significantly worse liver MFS (*P* = 0.028) compared to those without HS.

As obesity is reported to be a risk factor for HS and is significantly associated with BMI, the overweight and non-overweight groups were analyzed. As shown in Fig. 3A, for the outcome of overweight (BMI ≥25 kg/m^2^) patients, HS was not associated with liver MFS (*P* = 0.280). However, in the non-overweight group (BMI <25 kg/m^2^), HS was associated with a significantly worse prognosis in the liver MFS (*P* = 0.003) (Fig. 3B). Similarly, we analyzed the impact of age among the patients with HS. As shown in Fig. 4A, in the older age (age ≥60 years) group, the association of liver MFS was not observed in patients with or without HS. On the other hand, in patients with HS, younger age (age <60 years) was associated with a worse liver MFS (*P* = 0.028) (Fig. 4B).

### Prognostic factors for overall metastasis-free survival

**Figure 5:**
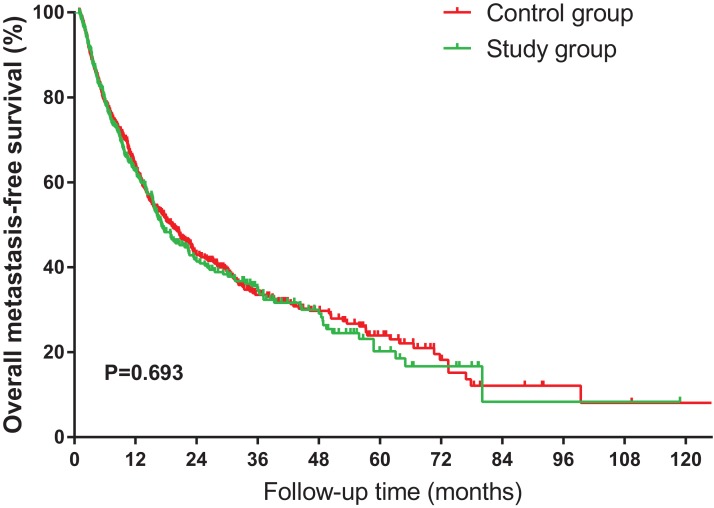
Kaplan–Meier curves of overall metastasis-free survival for NSCLC patients, stratified by HS.

The overall MFS was calculated from the date of the histological biopsy to the date of diagnosis of the first metastatic site, or to the last examination. As the overall MFS rates are shown in Fig. 5, we found there was no significant differences in overall MFS rates between the two groups (*P* = 0.693).

In the multivariate Cox regression analysis adjusted for all confounders, surgery (HR 0.53; 95% CI [0.44–0.64]; *P* < 0.001) and squamous histology (HR 0. 75; 95% CI [0.64–0.88]; *P* = 0.001) were found to be independent favorable factors for the overall MFS (Table 3). However, advanced T classification (T2, T3, and T4) and advanced N classification (N2 and N3) were associated with increased risks of overall metastasis in NSCLC patients.

**Table 3:** Univariate and multivariate analyses for overall metastasis.

Parameter	Univariate analysis		Multivariate analysis	
	HR (95% CI)	*P*-value	HR (95% CI)	*P*-value
Age ≥60 years	1.00 [0.87–1.16]	0.959	0.95 [0.82–1.10]	0.490
Gender. male	1.00 [0.86–1.17]	0.97	1.10 [0.94–1.29]	0.248
T category				
T1	1		1	
T2	2.23 [1.71–3.10]	<0.001	1.90 [1.40–2.57]	<0.001
T3	2.29 [2.12–3.99]	<0.001	1.92 [1.39–2.65]	<0.001
T4	3.29 [2.44–4.42]	<0.001	1.97 [1.45–2.67]	<0.001
N category				
N0	1		1	
N1	1.52 [1.11–2.11]	0.010	1.28 [0.92–1.77]	0.141
N2	3.19 [2.56–3.97]	<0.001	2.41 [1.92–3.02]	<0.001
N3	3.87 [3.07–4.88]	<0.001	2.52 [1.97–3.22]	<0.001
Histology squamous	0.78 [0.67–0.92]	0.002	0.75 [0.64–0.88]	0.001
ALT or AST ≥40 U/L	1.30 [1.04–1.62]	0.023	1.14 [0.90–1.44]	0.285
Hepatitis virus	1.26 [1.03–1.54]	0.026	1.21 [0.98–1.50]	0.071
Diabetes mellitus	1.0 2 [0.79–1.31]	0.898	1.09 [0.84–1.41]	0.520
BMI ≥25 kg/m^2^	1.13 [0.96–1.33]	0.152	1.05 [0.89–1.24]	0.584
Hepatic steatosis	1.03 [0.88–1.21]	0.693	0.99 [0.84–1.17]	0.895
Surgery	0.38 [0.32–0.49]	<0.001	0.53 [0.44–0.64]	<0.001
Chemotherapy	2.07 [1.52–2.82]	<0.001	1.22 [0.89–1.67]	0.224
Radiotherapy	1.43 [1.23–1.67]	<0.001	1.13 [0.97–1.33]	0.119

**Note:**

ALT, alanine aminotransferase; AST, aspartate aminotransferase; BMI, body mass index; HR, hazard ratio; CI, confidence interval.

## Discussion

Until now, the adverse factors for liver metastasis in patients with NSCLC have been far from well defined. To the best of our knowledge, the present study is the first-scale cohort study to analyze the effect of HS in NSCLC patients with no distant metastases. Among the patients in our study, the prevalence of the NAFLD was 21.8%, which is consistent with that of the NSCLC patients in China (Fan, Kim & Wong, 2017; Zhu et al., 2016) Moreover, the current study also found that HS was more common in younger (age <60 years) and male patients with NSCLC. Based on previous studies, NAFLD is common in middle-age male patients, and then declines after 50–60 years of age (Non-alcoholic Fatty Liver Disease Study Group et al., 2015; Wu et al., 2018). Consequently, this observed trend in our study is reasonable.

In this study, patients in the HS group had a significantly higher rate of liver metastasis compared with the control group. Further multivariate analyses suggested that HS was an independent risk factor for liver metastasis in patients with NSCLC, while HS had no significant effects on the development of overall metastasis. It is well-known that liver metastasis is a serious adverse prognostic factor for the overall survival of patients with NSCLC, but clinicopathological risk factors for the liver metastasis of NSCLC have not yet been well identified. Our findings provide evidence that HS is a significant risk factor in the development of liver metastasis, and may provide valuable ideas for future research.

Inflammation plays a key role in tumor progression, and it has been reported that changes in the microenvironment caused by inflammation contribute to metastasis (Coussens & Werb, 2002). Thus, we hypothesized that HS might be a risk factor for liver metastasis in patients with NSCLC, possibly by providing a favorable microenvironment for metastasis of tumor cells. In fact, several studies have reported that hepatic stellate cells can be activated by steatosis (Li et al., 2015; Mikuriya et al., 2015), which could play a key role in the progression of organizing and promoting metastasis, and organizing angiogenesis during the development of liver metastasis (Eveno et al., 2015; Matsusue et al., 2009). Furthermore, in a murine model, dietary fat increased metastatic tumor growth in the NAFLD microenvironment after the intrasplenic injection of colon cancer cells (VanSaun et al., 2009).

Additionally, copper has been found to contribute to the metastatic progression of cancers, which is a key component of several enzymes critical to remodeling the tumor microenvironment (Chan et al., 2017). Interestingly, serum copper levels were higher in lung cancer patients than in the healthy people (Zablocka-Slowinska et al., 2018). Although low copper levels may contribute to the pathogenesis of NAFLD (Tarantino et al., 2018), until now, there is no literature to report the effect of HS on copper metabolism. Moving forward, we hypothesize that HS inhibits the conversion of copper into compounds in liver tissue, as copper compounds were found to have promising anticancer properties (Shi et al., 2018). Consequently, further mechanistic studies are needed to study the HS microenvironment in NSCLC patients with HS.

The results of this study provide evidence of the effect of HS on the development of liver metastasis in patients with no distant metastatic NSCLC. Consequently, researchers in clinical trials of patients with NSCLC should pay attention to the prevalence rate of HS in different treatment groups, especially in China where the incidence of NAFLD is increasing rapidly (Lu et al., 2016). Thus, the management guidelines of NSCLC should consider patients with HS.

The current study has several limitations. First, as this was a retrospective study, there may be present some selection biases, and the incidence of liver metastasis was low. In addition, the loss to follow-up bias might have affected the outcomes due to the high mortality of NSCLC, and the sample size for this study was limited (408 patients in the study group). Therefore, these results need to be validated by a prospective study of a larger cohort. Second, NAFLD and liver metastasis were mainly evaluated via imaging examination and not validated by biopsy, which may lead to false positive diagnoses, although the results were evaluated separately by two radiologists. Pathological findings can be used to provide evidence in terms of microenvironment changes in the liver. Finally, due to the limitations of imaging results and laboratory data, we could not be able to study the impact of liver metastasis at different levels of HS.

## Conclusion

In this study, the data indicate that HS is a risk factor for liver metastasis in patients with no distant metastatic NSCLC. The finding of the current study offers meaningful suggestions regarding the potential crosstalk between metastases of tumor cells and the liver microenvironment, suggesting that more investigations should focus on the impact of HS on the progression of NSCLC. Further studies, including histopathologic investigations of liver tissue to confirm HS, should help clarify any potential relationship between HS and liver metastasis of NSCLC.

## Supplemental Information

10.7717/peerj.6612/supp-1 Supplemental Information 1 Raw data. followup1 was calculated from the day of the histopathological biopsy to the date of diagnosis of liver metastasis, or to the last imaging examination. followup2 was calculated from the date of the histological biopsy to the date of diagnosis of the first metastatic site, or to the last examination.

## Additional Information and Declarations

### Data Availability

The following information was supplied regarding data availability: The raw data are available in File S1.
